# Supplementary figures and images for: Distinctive features of the respiratory syncytial virus priming loop compared to other non-segmented negative strand RNA viruses
Source: PLoS Pathog. 2022 Jun 22;18(6):e1010451. doi: 10.1371/journal.ppat.1010451 (PMC9255747; doi:10.1371/journal.ppat.1010451)

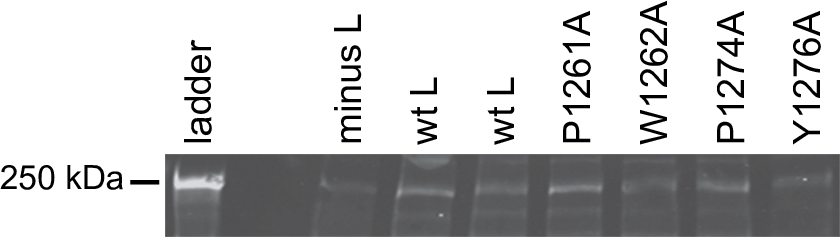

Supplement: S1 Fig — To quantify mutant L protein levels in transfected cells, the proteins were modified by adding two tandem FLAG tags to their N-termini. The FLAG-tagged L mutants were co-expressed with P protein in BSRT7/5 cells. At 48 h post transfection, cell lysates were analyzed by Western blotting using a FLAG-specific antibody. The image shown is representative of three independent experiments. Note that a much larger amount of L plasmid was required for protein detection by Western blotting than is optimal for minigenome assays and so we were unable to analyze protein levels in the same cells that were used for the minigenome RNA analysis shown in Fig 2, which employed lower levels of untagged L protein. A background band (visible in the minus L lane) complicated the analysis somewhat, but nonetheless, the results showed that all mutant proteins were expressed. The levels of the W1262A L protein were variable, and it was sometimes expressed at a lower level than that of the other mutants, suggesting that it might have been unstable at this temperature. However, we found that it was stable at 30°C and the levels of RNA synthesis for each of the L mutants at 30°C was similar as at 37°C (see S2 Fig). (TIF) [file ppat.1010451.s001.tif]

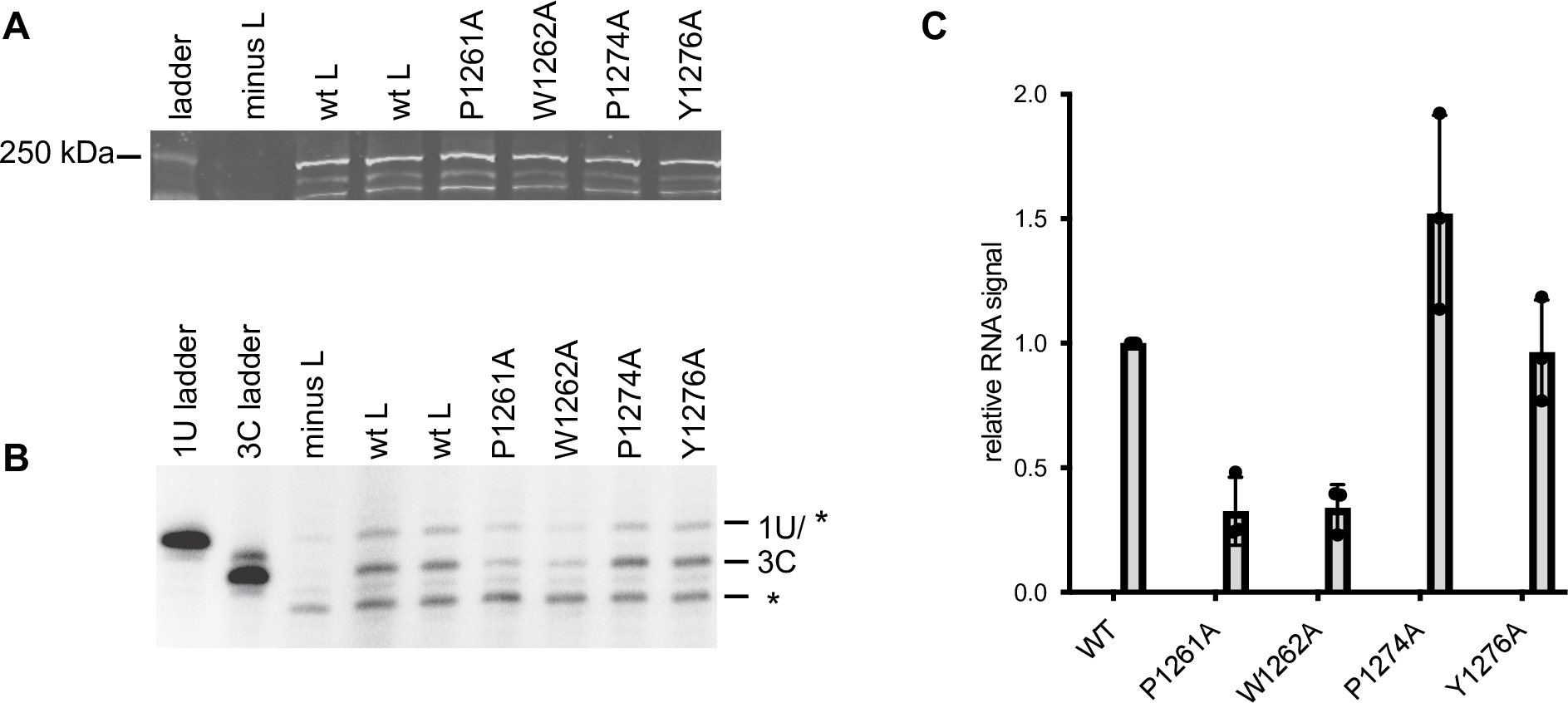

Supplement: S2 Fig — (A) L mutant proteins containing an N-terminal FLAG tag were expressed in mammalian cells at 30°C. At 48 h post transfection, cell lysates were analyzed by Western blot analysis using a FLAG-specific antibody. The image is representative of three independent experiments. (B) Analysis of RNA products generated from the 1U and 3C sites in a minigenome containing a le promoter (shown in Fig 2A) by mutant L polymerases, using the same transfection conditions as used in Fig 2, except that the cells were incubated at 30°C rather than 37°C. The asterisks indicate non-specific background bands. (C) Quantification of the 3C initiation products presented in panel B. The bars show the mean and standard deviation for three independent experiments (the data points for each experiment are shown). Unfortunately, a background band that migrated at the same position as the 1U product prevented accurate quantification of this product. However, quantification of the 3C product showed that L mutants P1274A and Y1276A yielded RNA at a similar level as wt L, whereas the P1261A and W1262A mutants yielded RNA from the 3C initiation site at approximately 35% of wt levels. These results are similar to those obtained at 37°C (Fig 2). (TIF) [file ppat.1010451.s002.tif]
